# Evidence for eosinophil and IL-17 mediated inflammation in allergic rhinitis

**DOI:** 10.1186/s12948-020-00117-6

**Published:** 2020-04-04

**Authors:** Kawa Amin, Sulaf Mosa Issa, Kosar Mohammad Ali, Muaid Ismiel Aziz, Huner Mohamed Hama Amieen, Jonas Bystrom, Christer Janson

**Affiliations:** 1 Department of Medicine and Microbiology/Immunology, College of Medicine, University of Sulaimani, Sulaimanyah, Iraq; 2 Department of Medical Science, Respiratory, Allergy and Sleep Research, Uppsala University, Uppsala, Sweden; 3 Otolaryngology Center, Sulaimani Teaching Hospital, Sulaimanyah, Iraq; 4 Expermiental Medicine and Rheumatology, William Harvey Research Institute, Barts & The London, Queen Mary, University of London, Charterhouse Square, London, EC1M 6BQ UK

**Keywords:** Allergic rhinitis, Immune system, ECP, IL-17, IL-33, IgE

## Abstract

**Background:**

The aim was to determine the level of inflammatory cytokines, eosinophil cationic protein and IgE in allergic rhinitis (AR) patients.

**Subjects and methods:**

Blood samples were taken from 88 AR patients and 88 healthy controls (HC). Each sample was analysed for eosinophil counts by flow cytometry, IgE by ECLIA, ECP, IL-17, and IL-33 by using ELISA test.

**Results:**

There was no significant difference between AR patients and the control group in age and gender. Levels of eosinophils, IgE, ECP, IL-17, IL-33 and the total symptom scores were significantly higher in AR patients than the HC (*P *=* 0.0001*). Serum ECP correlated with IL-17 (P = 0.041, r = 0.42), IL-33 (P = 0.0001, r = 080), and IgE levels (P = 0.017, r = 0.45) in the R patients. There was no correlation between IL-17 and IL-33. There was a correlation between symptom scores and eosinophils (P = 0.026, r = 0.52), and IgE (P = 0.001, r = 0.60) in the patients. No correlation was observed between symptom scores and ECP, IL-17, and IL-33 in the AR patient.

**Conclusions:**

Patients with AR have significant higher serum levels of ECP, IL-17, and IL-33 than healthy controls. This indicates that these markers could be used to in order to diagnose AR and to monitor disease. Inhibitory molecules to IL-17 and IL-33 may be considered as novel treatment strategies.

## Highlights

- There was relationship between serum ECP and IL-17, IL-33, IgE, and AR disease, respectively.
- A relationship was established between symptoms, blood eosinophil level and serum IgE level.
- IL-17 secreted by Th17 cell, acts upon various immune cells and might have a role in AR.
- IL-17 and IL-33, ECP may be considered as novel therapeutic targets in AR.

## Background

Allergic rhinitis (AR) is a disorder with increasing prevalence, already affecting 10–40% of the worldwide population [1, 2]. The symptoms can be non-specific and might be ignored by patients and physicians. Many individuals with disease do not report their complaints or seek treatment [3]. Accordingly, AR is considered a social problem that negatively affects patients’ quality of life and performance [4]. Recently, on the basis of frequency and duration of symptoms, AR has been categorized as either intermittent or persistent [5].

Characteristic for the onset of AR is an allergen specific IgE mediated activation of mast cells (MCs) in the nasal mucosa leading to mediator release and acute inflammation. This is followed, several hours after the initial allergen exposure by release of other inflammatory mediators, accumulation of inflammatory cells and a recurrence of symptoms. Low-grade underlying inflammation often remains in the mucosa [6].

Apart from MCs, other inflammatory immune cells and their mediators contribute to the inflammation in AR. Released mediators are associated with and attract a T-helper cell type 2 (Th2) cells and eosinophils that contribute to pathology [7]. MCs and eosinophils are considered to be central effector cells in the allergic reaction [8, 9]. Eosinophil cationic protein (ECP) is one of the mediators that upon activation is released from eosinophils during allergic diseases [10]. ECP possesses cytotoxic ribonuclease activity and have bactericidal and antiviral properties [11, 12]. The mechanism for killing target cells is by the formation of transmembrane pores and channels [10]. ECP has been quantified in bodily fluids, including serum, bronchoalveolar lavage (BAL) and nasal secretions of patients with allergic and other inflammatory diseases [13].

Interleukin-17 (IL-17) is a cytokine produced by Th17 (Th17) cells which is involved in host defences, the induction of inflammation and autoimmunity [14, 15]. Studies have indicated involvement of IL-17 in allergic responses [16]. However, the contribution of Th17 inflammation in AR has not been thoroughly assessed [17, 18].

Interleukin-33 (IL-33), is a member o the IL-1 family that is involved in the Th2 mediated immune response, host defences and allergic diseases [19]. The cytokine is produced by a variety of different cells such as macrophages, dendritic cells (DCs), MCs, fibroblasts, smooth muscle cells, endothelial- and epithelial cells [20].

Serum specific IgE (sIgE) antibody test and the skin prick test (SPT) are used to detecting AR [21]. IgE has been found to be highly specific, while SPT on the other hand is more sensitive [22]. The aim of this study was to analyze the level of the cytokines IL-17, IL-33, the eosinophil product ECP, in relation to IgE, in AR patients. We wanted to determine the usefulness of markers other than IgE and whether informative correlations between the inflammatory cells, their mediators and allergic symptoms existed. Such findings would indicate the mediators’ specific involvement and possible reciprocal relationship in AR disease.

## Subjects and methods

### Patients and setting of the study

For this study, 88 patients with AR and 88 healthy controls (HC) were recruited and compared. Samples from the subjects were collected at the laboratory of Public Health and Ali Kamal Health Center, Sulimani, Iraq. The complete blood count (CBC) and IgE level analysis were performed in the Public Health laboratory in Sulaimani (Table 1).

**Table 1:** Characteristics of both patients and healthy control—according to age, sex, symptoms, serum IgE and eosinophil

	Allergic rhinitis (n = 88)	Healthy subjects (n = 88)	*P* value
Age (year), (M ± SD)	33.2 ± 11.9	34 ± 11.6	0.363
Sex (female/male)	66/22	62 / 26	0.36 (ns)
Serum total IgE (IU/mL), (M+SD)	161 ± 221	35 ± 26	0.0001
Eosinophil (10^9^/L) (M+SD)	0.35 ± 2.9	0.23 ± 1.05	0.0004
Symptom	1.80 ± 0.40	0.01	0.0001

*P*-value <0.05 (significant)

*ns* Non-significant

### Inclusion and exclusion criteria

All of the AR patients were assessed by a consultant ear-, nose- and throat-specialist (ENT) according to the British Society for Allergy and Clinical Immunology (BSACI) criteria. AR was diagnosed based on history, symptoms, clinical examination and by the measurement of total serum IgE and CBC. The healthy control subjects were selected as having no history of allergy of any other chronic diseases. Patients, who had undergone treatment for allergies, before the blood was drawn, were excluded from this study.

### Ethical considerations

Ethical approval for the study was provided by The Ethic Committee of the college of Medicine, University of Sulaimani. Informed consent for the study was obtained from each patient and healthy individual that agreed to participate in the study.

### Specimens

Eight milliliter of venous blood were drawn from each participant in the study. Three milliliter were stored in an ethylene diamine tetra-acetic acid (EDTA) tubes used for an eosinophil test. The remaining 5 mL were transferred to a clot activator tube and centrifuged at 4500 RPM for 10 min. One aliquot of the serum was used for the IgE measurement and another was stored at − 80 °C until further use.

### Preparation and procedure of the different kit

Optical Flow Cytometry Kit for Detection of Eosinophils in Human Blood was used according to the manufacturer’s instructions (Orphee, France). IgE measurement in serum was performed using ECLIA kit according to the manufacturer’s instructions (Roch, USA). IL-17 and IL-33 was measured in serum using ELISA kits according to the manufacturer’s instructions (Abcam, UK). Human ECP (Eosinophil Cationic Protein) was measured in serum in accordance with directives provided by the manufacturer (Diagnostics Development, Uppsala, Sweden).

### Statistical analysis

Statistical analyses were performed using GraphPad version 5. Results are expressed as mean ± standard error (mean ± SE) or mean ± standard deviation (mean ± SD) as indicated. Data was analysed using the Student’s t-test or the Mann–Whitney-U test for comparison of two groups, or one-way analysis of variance for multiple comparisons (ANOVA). The Pearson product-moment correlation coefficient (r) was used to assess the relationship between cytokines, eosinophil and IgE levels. A *P*-*value* equal to or less than 0.05 was considered statistically significant (S). Otherwise, results from statistical analysis were considered non-significant (NS).

## Results

### Characteristics of patients and healthy control

This study included 88 patients with AR and as a control population, 88 apparently healthy subjects. Table 1 illustrates the basic characteristics of patients and the healthy controls. There were no difference in age comparing patients (33.2 ± 11.9 years of age) and HC (34.0 ± 11.6 years of age) and the male female ratio was in a similar range (female/male ration, patients: 66/22, healthy controls: 62/26). Patients had significantly higher level of IgE measurable in the serum than the healthy controls (161.0 IU/mL compared to 34.9 IU/mL, *P *= 0.0001). The clinical assessment showed high symptom score in the patients (1.80 ± 0.4, HC: 0.01, *P *= 0.0001). Of the patients, 80% had perennial and the remainder seasonal AR.

### Comparison

**Fig. 1:**
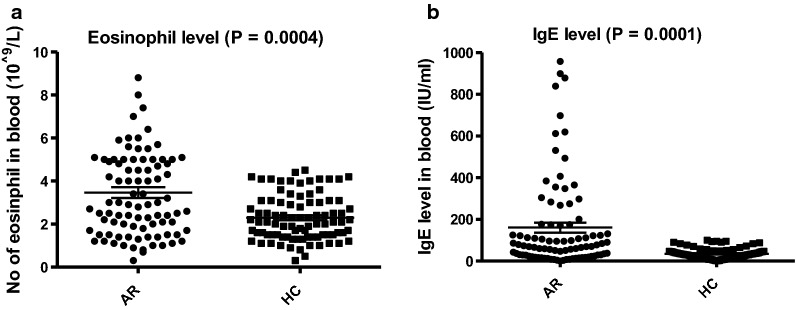
**a** Comparison of blood eosinophil counts (10^9^/L) in AR patients and HC subjects and **b** total IgE level (IU/mL) in serum of AR patients and HC. Error bars indicate median values

All AR patients’ had blood eosinophil count above the normal range. The patients’ blood eosinophil count was significantly higher that the HCs’ (see Table 1, 3.5 × 10^9^ compared to 2.3 × 10^9^, and Fig. 1a, *P *= 0.0004).

IgE was detectable in all patients serum and was considered elevated if above 100 IU/mL. While all the HC subjects had IgE levels within the normal range many of the patients had elevated levels (*P* = 0.0001, Fig. 1b).

**Fig. 2:**
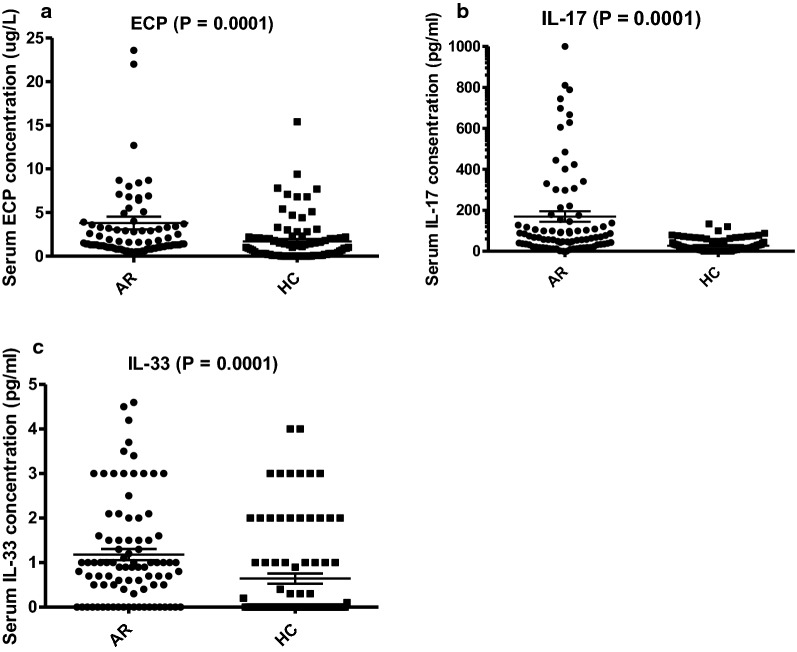
Difference in serum level of **a** ECP concentration (μg/L), **b** IL-17 (pg/mL), **c** IL-33 (pg/mL) comparing AR patients and HC. Error bars indicate median values

AR patients and healthy controls had detectable levels of ECP in their serum. Statistical analysis showed serum ECP levels in the AR patients serum were significantly higher than that of the HCs’ (3.80 ± 6.83 μg/mL and 1.70 ± 2.53 μg/mL respectively, *P* = 0.0001, Fig. 2a).

The cytokine IL-17 was detectable in 46 of the 88 AR patients but low or undetectable in HCs. A significant difference in the IL-17 serum levels was therefore found when comparing AR patients and healthy controls (169 ± 240 pg/mL and 27.0 ± 30.2 respectively, *P *= 0.0001, Fig. 2b).

Level of IL-33 was detectable in 67 of the 88 AR patients. Figure 2c shows the serum levels of IL-33 which is significantly higher in the AR group (1.18 ± 1.17 pg/mL) than the HC group (0.64 ± 1.10 pg/mL, *P* = 0.0001).

### Correlation

**Fig. 3:**
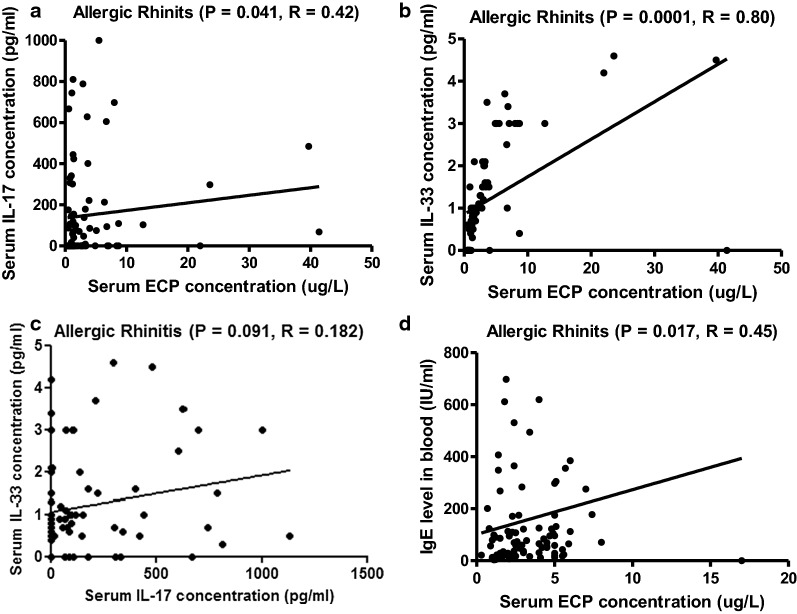
**a** Correlation between levels of ECP and IL-17, **b** between ECP and IL-33, **c** between IL-17 and IL-33 and **d** between ECP and IgE in serum of patients with AR

To assess whether there was a relationship between inflammatory mediators and cytokines present in serum of the 88 AR patients, correlation studies were performed. Figure 3a shows that a positive correlation emerged when comparing ECP and IL-17 levels (*P *= 0.041, R = 0.42). Furthermore as is shown in Fig. 3b, there was a positive correlation comparing ECP and IL-33 (*P *= 0.0001, R = 0.80). Statistical analysis showed on the other hand no correlation between IL-17 and IL-33 in the patients (*P *= 0.091, R = 0.182, Fig. 3c). Also level of ECP and IgE in serum of the patients was found to correlate (*P *= 0.017, R = 0.45).

Finally we wanted to determine whether there is a correlation between the patients’ symptoms and any of the inflammatory mediators or the cytokines. Such correlation was observed when comparing blood eosinophil levels or IgE with the patients’ symptoms (Table 2). ECP, IL-17 and IL-33 levels did not correlate with the patients symptom scores (Table 2).

**Table 2:** Correlation between symptoms and different markers in patients

	Eosinophil level in blood	IgE level in blood	ECP positive	IL-17 positive	IL-33 positive
Symptoms					
R-value	0.51	0.60	0.64	− 0.14	0.42
*P* value	0.026	0.0054	0.277	0.098	0.35
*P* value	*	**	ns	ns	ns

*ns* non-significant

*P-*value< 0.05 (significant)

*, ** *P* = 0.05, 0.01 compared with patients with allergic rhinitis

## Discussion

This study has shown that patients with allergic rhinitis experienced symptoms that relate to levels of IgE in serum and number of eosinophils in the blood. Furthermore by measurement of ECP and cytokines we have shown that the disease can be driven by eosinophils, IL-33 and a Th17 type inflammation. Our findings seem to suggest a reciprocal relationship between eosinophils and Th17 cells which is independent of IL-33.

The mean blood eosinophil counts in this study were significantly higher in AR patients than in the HC subjects. A similar finding was also observed by Beppu et al. [23]. Eosinophils are also present in healthy individuals, but in allergic diseases the release of various mediators such as IL-5, IL-3, and GM-CSF promotes the production and activation of the cells. Eosinophils do in turn secrete mediators that increase the allergic inflammation [24]. One of the eosinophil mediators is ECP which was also higher in the serum of patients with AR than in the healthy individuals in the present study. Similar results have been reported before [23, 25]. A review of data performed by Bystrom et al. confirmed that an elevated ECP concentration in the serum of AR patients is associated with human epithelial cell damage in the respiratory tract inflammatory disease [13].

In our study, levels of IL-17 in serum were significantly higher in AR patients compared to the HC group. Similar result has been reported in several other studies [39]. Milovanovic et al. showed in an experimental study that the involvement of IL-17/Th17 cells in allergy and they presented evidence that IL-17 enhances IgE production by human B cells in allergic patients suffering from asthma and atopic dermatitis compared with normal controls. They found that after the removal of the Th17 cells in vitro from the peripheral blood mononuclear cells (PBMCs) of allergic patients led to a decrease in IgE levels Addition of recombinant IL-17 to PBMCs on the other hand restores the level [26]. It is not known what is driving Th17 cell response in AR but it could be due to reduced number of regulatory T cells or antigen independent CD8+ suppressive T cells [27, 28]. High serum IL-17 levels may be associated with nasal inflammation, so targeting IL-17 (or augmentation of regulatory of suppressive T cells) could be a useful therapy in the treatment of AR disease [29].

Serum levels of IL-33 were significantly higher in the AR patients than in the HC group in our study. This is in accordance to the results presented in previous studies [30, 31]. Ketelaar et al. showed that IL-33 is present at very low levels in the serum of asthma patients using the ELISA technique, and this is in agreement with our finding; we did also detect low levels of the IL-33 in serum of patients with AR using the same technique [32]. A literature review by Rogala and Gluck concluded that IL-33 is a cytokine of interest but with both proinflammatory and anti-inflammatory properties. Hence, IL-33 is involved in Th2 mediated inflammatory responses in AR, but has protective effects in cardiovascular disease [33]. IL-33 has shown inhibitory properties in osteoporosis, a disease suggested to be promoted by IL-17. IL-17 is known to directly augmenting osteoclast development while IL-33 in was suggested to prevent such differentiation in favour of IL-4 production and alternatively activated macrophages [34]. In AR IL-33 can augment inflammation through activation of several types of cells including Th2 cells, MCs and eosinophils through its receptor ST2. We did also find a significant correlation between serum ECP and IL-33 levels in the AR patients. IL-33 can directly enhance eosinophils survival and degranulation [17], but no previous studies have proven a correlation between ECP and IL-33 in AR disease. These findings indicated that IL-33 and ECP play a key role in the nasal inflammation of AR patients and may be useful to assess the severity of the disease. Our finding indicates as well that the IL-33/ST2 pathway might serve as a therapeutic target to treat allergic inflammation [20].

In the current study, a significant correlation has been found between serum ECP and IL-17 levels in the AR patients. No previous studies have showed a correlation between ECP and IL-17 in patients with AR disease. In a Th2 skewed environment IL-17 can promote eosinophils survival and degranulation via GM-CSF [17]. In a Th1/Th17 skewed environment, IL-17 has been shown to promote autoimmunity and bone resorption but our findings demonstrate that in atopy IL-17 and ECP protein may augment nasal inflammation in AR patients [15, 17].

We also found a significant correlation between serum ECP and IgE levels in the AR patients. To our knowledge no previous studies have proven a correlation between ECP and IgE in AR disease. An explanation for this finding may be that the eosinophil degranulates and releases mediators such as ECP after contact with IgE through surface receptors in allergic diseases. In accordance with the findings of Voice et al. [35] there was however no significant correlation between serum IL-17 and IL-33 levels in the AR group.

We found a significant correlation between symptom scores and eosinophil count. Apar et al. also showed a significant correlation between eosinophil count and the symptom severity score for AR [36] and Chen et al. observed a correlation of eosinophil count with the symptom severity score of perennial allergic rhinitis (PAR) [37]. In our study a high proportion, 80% of the patients had PAR. We did also find a significant correlation between IgE levels and symptom scores. AR symptoms, either sneezing-rhinorrhea or nasal obstruction, might therefore occur due to the release of allergic mediators, such as histamine when allergens cross-links IgE bound to the surface of MCs in AR patients [8]. Moreover, the study by Chen et al. shows that levels of IgE have been correlated with symptom severity score in PAR patients [37].

No statistically significant correlation was found between ECP, IL-17, IL-33 levels and symptom scores. This result is in agreement with one previous study [38]. In contrast to our results Chen et al. found the symptom severity score was correlated with serum ECP levels in PAR patients [37]. Our results are also in disagreement with those reported by Ciprandi and Gluck where a significant correlation between symptom severity score and IL-17 and also IL-33 was found [31, 39, 40].

## Conclusion

In conclusion, patients with AR have higher serum levels of ECP, IL-17, and IL-33 than healthy controls. This indicates that these markers could be used to in order to diagnose AR patients and also that inhibitory molecules blocking IL-17 and IL-33 might be considered as future therapeutic targets.
